# The Impact of Text Messaging on Medication Adherence and Exercise Among Postmyocardial Infarction Patients: Randomized Controlled Pilot Trial

**DOI:** 10.2196/mhealth.7144

**Published:** 2017-08-03

**Authors:** Avinash Pandey, Alexis A Krumme, Tejal Patel, Niteesh K Choudhry

**Affiliations:** ^1^ University of Western Ontario London, ON Canada; ^2^ Division of Pharmacoepidemiology and Pharmacoeconomics Department of Medicine Brigham and Women’s Hospital and Harvard Medical School Boston, MA United States; ^3^ Center for Healthcare Delivery Sciences Department of Medicine Brigham and Women’s Hospital and Harvard Medical School Boston, MA United States; ^4^ School of Pharmacy University of Waterloo Waterloo, ON Canada

**Keywords:** medication adherence, cardiac rehabilitation, exercise, randomized controlled trial

## Abstract

**Background:**

Adherence to evidence-based therapies such as medications and exercise remains poor among patients after a myocardial infarction (MI). Text message reminders have been shown to improve rates of adherence to medication and exercise, but the existing studies have been of short duration.

**Objective:**

Two single-center randomized controlled pilot trials were conducted to evaluate the impact of text message reminders over 12 months on adherence to cardiac medications and exercise among patients receiving cardiac rehabilitation after hospitalization for MI.

**Methods:**

In the medication adherence trial, 34 patients were randomized to receive usual care alone or usual care plus daily text message reminders delivered at the time of day at which medications were to be taken. In the exercise adherence trial, 50 patients were randomized to receive usual care alone or usual care plus 4 daily text messages reminding them to exercise as directed.

**Results:**

The text message reminders led to a mean 14.2 percentage point improvement in self-reported medication adherence over usual care (*P*<.001, 95% CI 7-21). In the exercise trial, text message reminders resulted in an additional 4.2 days (*P*=.001, 95% CI 1.9-6.4) and 4.0 hours (*P*<.001, 95% CI 2.4-5.6) of exercise per month over usual care and a nonsignificant increase of 1.2 metabolic equivalents (METS; *P*=.06) in exercise capacity as assessed by a BRUCE protocol at 12 months.

**Conclusions:**

Text message reminders significantly increased adherence to medication and exercise among post-MI patients receiving care in a structured cardiac rehabilitation program. This technology represents a simple and scalable method to ensure consistent use of evidence-based cardiovascular therapies.

**Trial Registration:**

Clinicaltrials.gov NCT02783287; https://clinicaltrials.gov/ct2/show/NCT02783287 (Archived by WebCite at http://www.webcitation.org/6sBnvNb05)

## Introduction

Acute myocardial infarction (MI) remains a leading cause of death and disability worldwide [1]. The use of evidence-based therapies, including exercise and medications, has contributed to substantial reductions in cardiovascular morbidity and mortality [2]. Whereas the prescription of these highly effective therapies is now nearly universal, many patients do not adhere to their medications or exercise regimens over the long term, which in turn is associated with negative consequences for cardiovascular outcomes and spending [3-5].

Although many factors are associated with such gaps in care, simple forgetfulness has been identified as a key contributor [6-8]. Strategies to address this barrier include improvements to pillboxes and bottles, including manual and electronic reminder systems; however, only a few have been rigorously evaluated and, in some cases, their complexity, cost, and the need for patients to purchase and set them up to use them may limit their ultimate value [9-13].

With the widespread use of mobile phones, including the recent increase in uptake among elderly patients, text message–based reminders may represent a potentially effective avenue for intervention [14-16]. Although some clinical benefit from text message reminders has been established in improving both medication and exercise adherence, there is little evidence about the effectiveness of this strategy for patients recently discharged after MI [17-20], which represents a particularly high-risk patient subgroup [21]. The few studies that do exist have been of limited duration, with the longest study among post-MI patients being of an 8-week duration only. Accordingly, we sought to assess the impact of structured daily text message reminders on adherence to post-MI medications and exercise in two randomized controlled pilot trials.

## Methods

To assess the impact of text message reminders on adherence to medications and exercise, we conducted two single-center, open-label, randomized controlled pilot trials in patients recently discharged from hospital after MI. This research was approved by the University of Waterloo Research Ethics Committee (20980) and is registered with clinicaltrials.gov (NCT02783287).

The trials were conducted at Cambridge Cardiac Rehab in Ontario, Canada, which enrolled consecutive patients aged 18 years and older, who within the preceding 2 weeks had been discharged from hospital after MI and enrolled in a structured cardiac rehabilitation program. In the medication adherence trial, we included patients who were receiving treatment with medications from all four of the following classes: antiplatelets, beta-blockers, angiotensin converting enzyme inhibitors-I or angiotensin receptor blockers, and 3-hydroxy-3-methylglutaryl- coenzyme reductase enzyme inhibitors (statins). As the trial involved daily text messaging, patients taking medications in dosing regimens of more than once daily were excluded. In the exercise adherence trial, we included patients who had been prescribed an exercise regimen comprising 45 min of exercise 5 days a week. In both trials, individuals without a mobile phone, those who were unable to read and write in English or provide informed consent, or those who were incarcerated were excluded.

In both trials, following recruitment and written informed consent, we collected demographic information, including age, gender, medical diagnosis, medication list, and educational status. Participants were randomized using a Web-based random number generator in a 1:1 ratio to intervention or control. Patients were not permitted to participate in both trials.

In the medication adherence trial, patients randomized to intervention received daily text messages at the time they preferred to take their medications. The text messages simply indicated that patients should remember to take their medications and contained no identifiable information such as medications names or classes. For example, patients who took their medication in the morning received a text message that read, “Please remember to take your morning medications now.” Patients randomized to the control arm of the study did not receive text reminders. Automated text message reminder software was developed with Microsoft Small Basic, a simplified version of Microsoft Visual Basic, and did not utilize bidirectional contact.

For the exercise adherence trial, patients randomized to the intervention group were sent text message reminders 4 times daily, at 7:30 am, 12:00 pm, 6:00 pm, and 9:00 pm. The text message reminders simply read, “Please remember to exercise for 30 minutes today.” These timings were identified as common timings for exercise by the staff of the cardiac rehabilitation center where the study was conducted. Patients randomized to the control arm of the study did not receive any text message reminders.

All patients in both trials participated in the structured outpatient cardiac rehabilitation program in which they were enrolled. The program comprised 1 week of education about diet, proper exercise technique, heart disease and MI, smoking cessation, and stress management and was followed by 2 sessions per week of on-site exercise under the supervision of kinesiologists and nurses. The study protocol was conducted with the help of the staff at the study site. Cardiac rehabilitation nurses and kinesiologists distributed and collected logbooks and enrolled patients into the text message reminder system. After completing the initial 3 months of the program, patients continued their rehabilitation at home but were instructed to return for a follow-up assessment at 1 year. In both trials, patients were followed up for up to 12 months, including the 3 initial months of the program, and patients and their health care providers were aware of the arm to which they had been randomized.

In the adherence trial, all study participants were asked to use a logbook to record the name and timing of the medications they had taken on a daily basis. The logbooks were based on the standard logbooks used by the cardiac rehabilitation center as suggested by The Canadian Association of Cardiovascular Prevention and Rehabilitation. The logbooks had 1 page per day, with separate rows for different times of the day. Patients were instructed not to log anything if they missed a medication or did not exercise on a specific day. Missing entries were interpreted as a missed dose. Logbooks were collected monthly. From these logs, absolute medication adherence was calculated as the percentage of total prescribed doses that were actually taken each month. For example, if a patient took 27 out of the 30 prescribed doses for a month, they were deemed to be 90% adherent. The primary outcome for this trial was average adherence over 12 months, calculated as the mean of each of the 12 monthly measurements of the percentage of days covered (PDC). The secondary outcome was “full adherence,” which was a measure of whether average adherence over the 12 months of follow-up was greater than 80%, which in turn represented a level of use above which patients with coronary artery disease benefit from statins and the threshold used by most quality measures [21-23].

In the exercise trial, all participants were asked to log the time when they started and finished exercising every day. Logbooks were collected on a monthly basis. The primary outcome for this trial was the average number of days per month that a patient exercised over 12 months, calculated as the mean of each of the 12 monthly measurements of the days per months exercised. Secondary outcomes included the average number of hours of exercise per month and cardiopulmonary fitness as assessed by a BRUCE protocol 12 months after randomization [24,25]. Cardiopulmonary fitness was evaluated with an exercise stress test conducted according to the BRUCE protocol, which is the most widely used method employed in routine clinical practice. In this approach, patients walk on a treadmill and the speed and inclination are adjusted based on a preset schedule. Patients walk until they are unable to continue or are instructed to stop by the technician conducting the test. The duration of time that elapses before stopping is then converted to metabolic equivalents (METS) based on standard calculations.

Sample sizes for the trials were estimated based on the results of two preparatory studies conducted at the Cambridge Cardiac Care Center. The medication adherence study was a 2-month crossover study of 30 patients who were recently discharged from hospital after admission for coronary artery disease; these patients were randomized to receive text message reminders for 1 month and to receive no text message reminders the other month. The exercise adherence study was a 2-month parallel group study of 16 post-MI patients who were randomized to either receive daily text message reminders or not to receive daily text message reminders for 2 months. The medication adherence pilot found a 10.8 percentage point (standard deviation [SD] 8.9) increase in adherence from text message reminders. On the basis of this increase in adherence, and assuming an alpha of .05, we estimated that we could achieve 80% power with a sample size of at least 32 patients. The exercise adherence pilot trial found an increase of 10.3 (SD 18.7) days per month of exercise; on the basis of this increase and assuming an alpha of .05, we estimated that we could achieve 80% power with a sample size of 50 patients.

All analyses were performed based on the intention-to-treat principles. We calculated means and frequencies of prerandomization variables separately by study arm. For the medication adherence trial, we evaluated the impact of text messaging on average adherence and optimal adherence using linear and logistic regression, respectively. For the exercise adherence trial, all outcomes were evaluated using linear regression. After performing these analyses, we ran multivariable models adjusting for characteristics that were imbalanced by chance at baseline.

We additionally conducted hypothesis-generating subgroup analyses on the following factors: sex, age >65 years versus ≤65 years, postsecondary education versus up to postsecondary education, and self-reported depression versus no depression at baseline. Specifically, we evaluated whether model-based interaction terms were statistically significant for the primary outcome. We used Statistical Analysis System version 9.4 for all statistical analyses.

## Results

### Medication Adherence

**Figure 1:**
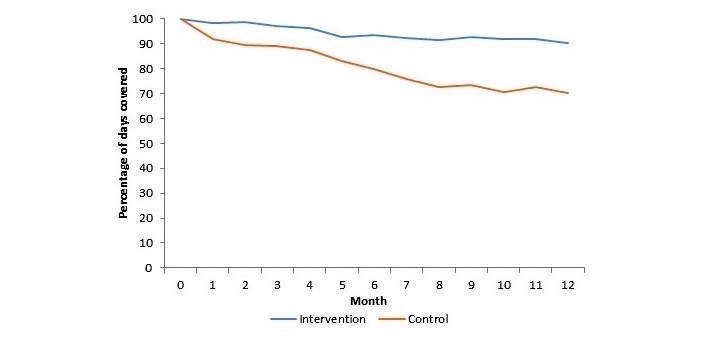
Monthly percentage of days covered (PDC) by study arm.

We screened a total of 90 patients discharged from hospital after acute MI, of whom 56 (63%) did not meet study inclusion criteria or declined to participate (Figure 1). The remaining 34 were randomly assigned to receive usual care alone (control) or usual care plus text message reminders (intervention). One control patient withdrew from the study during the first month of the study. Baseline characteristics of the study participants are shown in Table 1. Intervention subjects were slightly older, more likely to be smokers, taking more medications at baseline, and female. Baseline adherence before the beginning of the study was not measured. At month 1, patients in the control group achieved 90% medication adherence.

The impact of text messaging on medication adherence is shown in Figure 1 and Table 2. The average medication adherence during follow-up for patients randomized to usual care was 80% (95% CI 73-86). The mean difference in PDC between the text message and control groups was 14.2 percentage points (95% CI 7-21, *P*<.001; Table 2). All intervention patients were optimally adherent to their prescribed medications during follow-up compared with 50% (8/16) of control patients (*P*<.001). The results remained unchanged in a multivariable model adjusting for differences in sex, the only characteristic that was statistically significantly imbalanced at baseline.

**Table 1:** Baseline characteristics.

Characteristic				Adherence trial					Exercise trial		
			Text message reminders (N=17)		Usual care (N=16)		Text message reminders (N=25)			Usual care (N=25)	
**Demographic**											
	Age in years, mean (SD)			64.6 (11.5)		62.1 (11.0)		64.3 (10.7)			63.7 (9.6)
	Male gender, n (%)			6 (35)		14 (88)		15 (60)			11 (44)
	**Education^a^**										
		Secondary school, n (%)	10 (67)		8 (50)		17 (71)			16 (64)	
		Postsecondary school, n (%)	5 (33)		8 (50)		7 (29)			9 (36)	
**Clinical characteristics**											
	Cancer, n (%)			1 (6)		1 (6)		2 (8)			1 (4)
	COPD^b^, n (%)			3 (18)		3 (19)		4 (16)			5 (20)
	Dementia, n (%)			3 (18)		2 (13)		5 (20)			4 (16)
	Depression (self-report), n (%)			6 (35)		6 (38)		12 (48)			10 (40)
	Diabetes, n (%)			6 (35)		7 (44)		10 (40)			8 (32)
	Heart failure^c^, n (%)			-		-		10 (40)			9 (36)
	Chronic kidney disease, n (%)			2 (12)		3 (19)		6 (24)			5 (20)
	Smoker, n (%)			7 (41)		4 (25)		10 (40)			9 (36)
	Stroke, n (%)			4 (24)		1 (6)		4 (16)			3 (12)
	Baseline BRUCE^c^, mean (SD)			-		-		5.0 (1.7)			5.1 (1.9)
	Number of medications^d^, mean (SD)			10.1 (4.5)		8.0 (5.2)		10.5 (4.6)			9.0 (6.1)

^a^2 patients missing from Adherence trial, intervention arm; 1 patient missing from Exercise trial, intervention arm.

^b^COPD: chronic obstructive pulmonary disease.

^c^Not collected for Adherence trial.

^d^1 patient missing from Adherence trial, control arm.

**Table 2:** Impact of text messaging on study outcomes.

Trial and outcome			Parameter		Text message reminders (95% CI)		Usual care (95% CI)		Difference (95% CI)		*P* value^a^
**Medication adherence**											
	Primary	Average percentage of days covered during follow-up		94 (92-96)		80 (73-86)		14 (7-21)		<.001	
	Secondary	Percentage (N) fully adherent during follow-up		100 (17)		50 (8)		50		<.001^b^	
**Exercise adherence**											
	Primary	Mean days of exercise per month during follow-up		17.2 (16.0-18.5)		13.1 (11.1-15.0)		4.2 (1.9-6.4)		.001	
	Secondary	Mean hours of exercise per month during follow-up		12.5 (11.5-13.6)		8.5 (7.3-9.8)		4.0 (2.4-5.6)		<.001	
		Cardiopulmonary fitness at month 12 (METS)^c^		7.4 (6.5-8.3)		6.2 (5.2-7.2)		1.2 (−0.1 to 2.5)		.06	

^a^Satterthwaite method, except where noted.

^b^Fisher exact test.

^c^METS: metabolic equivalents, where 1 MET is equal to a whole-body oxygen consumption of 3.5 mL O_2_/kg/min.

**Table 3:** Subgroup analysis.

Trial and subgroup			Text message reminders (N)		Usual care (N)		Absolute difference		*P* value^a^
**Adherence outcome: percentage of days covered**									
	Male	93 (6)		80 (14)		13		.95	
	Female	94 (11)		81 (12)		13		.95	
	Age >65 years	91 (7)		73 (8)		18		.01	
	Age ≤65 years	96 (10)		85 (8)		11		.01	
	>12 years of education	94 (5)		82 (8)		12		.60	
	<12 years of education	93 (10)		77 (8)		16		.60	
	Depression (self-report)	95 (6)		79 (6)		16		.95	
	No depression	94 (11)		80 (10)		14		.95	
**Exercise outcome: days of exercise per month**									
	Male	16.8 (15)		11 (11)		5.8		.38	
	Female	19.2 (10)		10.5 (14)		8.7		.38	
	Age >65 years	18.2 (12)		12 (11)		6.2		.15	
	Age ≤65 years	16 (13)		14.2 (14)		1.8		.15	
	>12 years of education	17.5 (7)		13.9 (9)		3.6		.74	
	<12 years of education	17.1 (17)		12.6 (16)		4.5		.74	
	Depression (self-report)	18.4 (12)		12.6 (10)		5.8		.31	
	No depression	15.8 (13)		12.7 (15)		3.1		.31	

^a^Regression model-based interaction term.

Adherence was consistently higher for patients randomized to receive text messages across all subgroups but was statistically significantly only for age (Table 3). Text messages increased adherence by an average of 18% among patients ≥65 years of age compared with 11% among patients younger than 65 years (*P* value for interaction .009).

### Exercise Adherence

**Figure 2:**
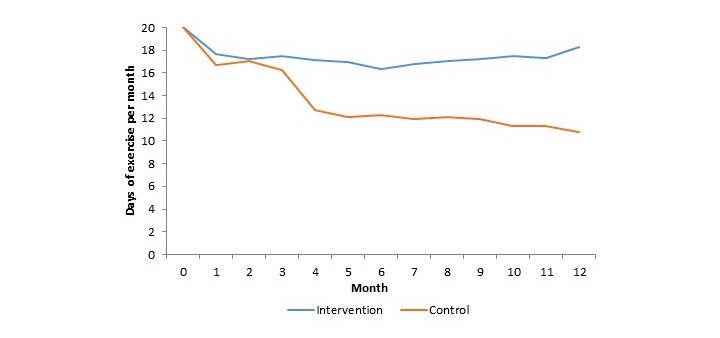
Monthly days of exercise by study arm.

We screened a total of 92 patients discharged from hospital after an acute MI (Figure 2). Of these, 42 were ineligible to participate in the study. The remaining 50 patients were randomized to either receive usual care or usual care in addition to text message reminders.

Baseline characteristics of the study participants are shown in Table 1. As in the medication adherence trial, intervention subjects tended to be older, more likely to smoke, and, on average, taking more medications at baseline. Additionally, intervention subjects were more likely to be female but were similar to controls with respect to cardiopulmonary fitness. Baseline exercise behavior before the beginning of the study was not observed. At month 1, patients in the control group exercised on an average of 16.7 days per month.

The impact of text messaging on exercise adherence is shown in Figure 2 and Table 2. The patients assigned to usual care exercised on an average of 13.1 days per month. In the text message group, the average number of days of exercise per month increased by 4.2 days (95% CI 1.9-6.4) to 17.2 days (*P*<.001). In multivariable models adjusting for imbalances in baseline characteristics, the results remained unchanged.

Patients receiving text messages also exercised an additional 4.0 hours per month (*P*<.001) and had a nonstatistically significant increase of 1.2 METS (*P*=.06) of exercise capacity. In subgroup analyses, days of exercise per month were consistently higher across all strata of patients randomized to the intervention arm (Table 3).

## Discussion

### Principal Findings

In this pair of randomized controlled pilot trials of text message reminders delivered to post-MI patients participating in cardiac rehabilitation, we found significant improvement in both adherence to medications and the frequency and duration of exercise over 12 months of follow-up as measured by patient self-report. In addition, the secondary outcome of the trial focusing on exercise adherence showed a nonsignificant trend to improved cardiopulmonary fitness as measured by exercise stress testing.

Several studies have demonstrated the potential benefits of external reminders, including text messaging, for improving medication adherence [17,26]. A recent meta-analysis of 16 randomized studies of adult patients with chronic disease, including coronary artery disease, HIV, and epilepsy, found that the odds of full adherence was more than doubled for those who received text messages [17]. Of the studies included, only 3 explicitly studied patients taking medications for secondary prevention after acute coronary syndrome, of which only 2 were ultimately included in the meta-analysis [19,20]. Both studies followed patients for only 30 days, sending daily personalized reminders to patients in the intervention arms to take their cardiovascular medications. Moreover, neither of these studies was conducted within the context of a comprehensive post-MI cardiac rehabilitation program, in which approximately 40% of patients post-MI in the United States enroll [27-29], and which could conceivably have augmented or extinguished the impact of text message reminders. In contrast, our study, despite being small in size and conducted at a single center, found sustained improvements in medication-taking from text message reminders delivered in addition to cardiac rehabilitation, as compared with cardiac rehabilitation alone that persisted over the course of 12 months of follow-up.

Several studies have demonstrated the beneficial effects of text message reminders on exercise adherence among patients with heart disease [26,30,31]. For example, the TEXT-MI trial enrolled patients with documented cardiovascular disease and randomized them to usual care or to receive text messages that provided advice, motivational reminders, and support to change a wide range of lifestyle behaviors, one of which was physical activity [26,30,31]. This trial found clinically meaningful and statistically significant improvements in exercise, low-density lipoprotein cholesterol, blood pressure, body mass index, and smoking even though follow-up ended after 6 months. A few existing studies have specifically enrolled patients in the immediate post-MI setting or have been conducted concurrently with cardiac rehabilitation. Frederix et al [32] studied the impact of augmenting cardiac rehabilitation with a “tele-rehabilitation” program in which emails and texts were sent by a coach based upon patient’s individual accelerometer data. The intervention resulted in significant improvements in physical fitness and quality of life over 24 weeks of follow-up. In contrast, our intervention relied on fully automated messages, without the need to receive and process accelerometer data, and followed patients for 12 months. The improvements in self-reported exercise adherence translated to higher exercise tolerance, as measured by the BRUCE protocol exercise stress test administered at 12 months. Although these results did not reach statistical significance, they demonstrate a strong long-term trend that bears confirmation in a larger study.

There are many similarities between medication adherence and exercise adherence behaviors that suggest why a highly similar intervention may have been effective for both behaviors. In specific, medication and exercise adherence require daily engagement and have multiple barriers to their performance, including motivation, literacy or knowledge, or simple forgetfulness. Moreover, studies of text messaging to improve medication adherence or exercise adherence have reported high patient satisfaction, with the vast majority of patients reporting that the short message service messages were useful and easy to understand [19,26,31].

A greater drop-off in the days of exercise per month was noted in the control group at month 3. This corresponds to the end of the on-site cardiac rehabilitation program. For the first 3 months of cardiac rehabilitation, patients exercised on-site for 2 sessions per week. Following this, patients continued to exercise at home but did not exercise on-site. No similar drop-off was noted in the text message arm of the study.

In our subgroup analyses, we observed consistent effects of texting on medication and exercise adherence. Although we had hypothesized a priori that access and knowledge of the technology may have been a barrier to effective use of this intervention among the elderly, our observations revealed the contrary. These results are consistent with studies that found other electronic interventions to be as or more effective among older individuals [33,34]. This being said, more research is required to confirm these results in larger and more diverse patient populations.

Patients were surveyed following the end of the study to discuss their experience with daily text message reminders. The majority reported that these reminders were helpful in maintaining adherence to their therapies and did not find them to be overly intrusive. They reported their preference for the text messages over alarms, which could be turned off more easily. Patients also reported liking that the reminders came from an outside source and likened them to calls from a nurse or other health care provider. More formalized polling of patient participants in future studies may provide further insight into patient experience with reminder systems such as the one examined in this study.

### Limitations

There are several limitations to our trials. First, both trials had small sample sizes and were conducted at a single cardiac rehabilitation facility in Ontario, Canada. Future studies with larger sample sizes could seek to evaluate other end points such as hospital readmissions. Data collected in this study were largely self-reported, although this has been the primary mode of outcome evaluation in the majority of existing studies evaluating text reminders on medication adherence [17]. The fact that the improvement in exercise adherence we observed was correlated with objective improvements in aerobic fitness measured by stress testing suggests that our outcome measurement was reliable and any mismeasurement was nondifferential with respect to the exposure. Our medication adherence trial included only patients on once-a-day regimens of the four classes of medications studied. Whereas all four medication classes we studied are widely available with this dosing strategy, our results may not be generalizable to all post-MI patients. Additionally, most of the patients in the study had one or more chronic diseases and were taking medications for these diseases. The message sent to patients reminding them to take their medications read, “Please remember to take your morning medications now.” Although we expect this nonspecific messaging would have encouraged patients to take all of their therapies as prescribed, we only evaluated adherence to the four post-MI medications. Also, the long follow-up of the study during which no one dropped out and which required patients to complete logbooks on a daily basis may limit generalizability to other patient groups. In both trials, logbooks were used to measure medication and exercise adherence because this method, previously validated, was already in use at the cardiac rehabilitation center where we conducted the study, and we hoped to integrate study procedures into the cardiac rehabilitation program. It is possible that patients may have taken a medication or exercised but forgotten to log this in the logbook; however, we do not believe that this has significantly impacted our results [35]. Finally, there were baseline imbalances in the treatment arms in potentially important characteristics, notably sex, although our overall results remained unchanged in multivariable and subgroup analyses. Further research should be conducted in larger and more diverse populations.

### Conclusions

In conclusion, structured text message reminders were found to significantly improve adherence to both medications and exercise. Although further research is required to validate our results in larger and more diverse settings, text messaging appears to represent a simple and scalable strategy for improving adherence to medications and exercise among post-MI patients.

## Abbreviations

METS: metabolic equivalents
MI: myocardial infarction
PDC: percentage of days covered

## Appendix

Multimedia Appendix 1 CONSORT-EHEALTH checklist (V1.6.1).
