# Author Correction: The Impact of Text Messaging on Medication Adherence and Exercise Among Postmyocardial Infarction Patients: Randomized Controlled Pilot Trial

**DOI:** 10.2196/mhealth.9254

**Published:** 2019-04-03

**Authors:** Avinash Pandey, Alexis A Krumme, Tejal Patel, Niteesh K Choudhry

**Affiliations:** 1 University of Western Ontario London, ON Canada; 2 Division of Pharmacoepidemiology and Pharmacoeconomics Department of Medicine Brigham and Women’s Hospital and Harvard Medical School Boston, MA United States; 3 Center for Healthcare Delivery Sciences Department of Medicine Brigham and Women’s Hospital and Harvard Medical School Boston, MA United States; 4 School of Pharmacy University of Waterloo Waterloo, ON Canada

The corresponding author for “The Impact of Text Messaging on Medication Adherence and Exercise Among Postmyocardial Infarction Patients: Randomized Controlled Pilot Trial” (JMIR MHealth UHealth 2017 Aug 3;5(8):e110) has been changed from Alexis Krumme to Niteesh Choudhry, whose contact information is as follows:

Niteesh K Choudhry, MD, PhD

Brigham and Women’s Hospital, Harvard Medical School

1620 Tremont Street, Suite 3030

Boston, MA, 02120

Phone: 1 617 278 0930

Fax: 1 617 232 8602

Email: nkchoudhry@bwh.harvard.edu

Additionally, middle initials have been added to the following authors’ names:

Alexis A Krumme

Niteesh K Choudhry

The correction will appear in the online version of the paper on the JMIR website on April 3, 2019, together with the publication of this correction notice. Because this was made after submission to PubMed, PubMed Central, and other full-text repositories, the corrected article also has been resubmitted to those repositories.

